# Characterization of tracheobronchomalacia in infants with hypophosphatasia

**DOI:** 10.1186/s13023-020-01483-9

**Published:** 2020-08-06

**Authors:** Raja Padidela, Robert Yates, Dan Benscoter, Gary McPhail, Elaine Chan, Jaya Nichani, M. Zulf Mughal, Charles Myer, Omendra Narayan, Claire Nissenbaum, Stuart Wilkinson, Shanggen Zhou, Howard M. Saal

**Affiliations:** 1 Department of Paediatric Endocrinology, Royal Manchester Children’s Hospital and Faculty of Biology, Medicine and Health, University of Manchester, Manchester, UK; 2 McMaster University, Hamilton, ON Canada; 3 Cincinnati Children’s Hospital Medical Center and University of Cincinnati College of Medicine, Cincinnati, OH USA; 4 Great Ormond Street Hospital, London, UK; 5 University of Manchester, Manchester, UK; 6 Covance, Inc., Princeton, NJ USA

**Keywords:** Hypophosphatasia, Tracheobronchomalacia, Respiratory failure, Respiratory support, Asfotase alfa

## Abstract

**Background:**

Perinatal and infantile hypophosphatasia (HPP) are associated with respiratory failure and respiratory complications. Effective management of such complications is of key clinical importance. In some infants with HPP, severe tracheobronchomalacia (TBM) contributes to respiratory difficulties. The objective of this study is to characterize the clinical features, investigations and management in these patients.

**Methods:**

We report a case series of five infants with perinatal HPP, with confirmed TBM, who were treated with asfotase alfa and observed for 3–7 years. Additionally, we reviewed respiratory function data in a subgroup of patients with perinatal and infantile HPP included in the clinical trials of asfotase alfa, who required high-pressure respiratory support (positive end-expiratory pressure [PEEP] ≥6 cm H_2_O and/or peak inspiratory pressure ≥18 cm H_2_O) during the studies.

**Results:**

The case series showed that TBM contributed significantly to respiratory morbidity, and prolonged respiratory support with high PEEP was required. However, TBM improved over time, allowing weaning of all patients from ventilator use. The review of clinical trial data included 20 patients and found a high degree of heterogeneity in PEEP requirements across the cohort; median PEEP was 8 cm H_2_O at any time and some patients presented with high PEEP (≥8 cm H_2_O) over periods of more than 6 months.

**Conclusion:**

In infants with HPP presenting with persistent respiratory complications, it is important to screen for TBM and initiate appropriate respiratory support and treatment with asfotase alfa at an early stage.

**Trial registration:**

ClinicalTrials.gov numbers: NCT00744042, registered 27 August 2008; NCT01205152, registered 17 September 2010; NCT01176266, registered 29 July 2010.

## Background

Hypophosphatasia (HPP) is a rare, systemic, inherited, metabolic disease caused by deficient activity of the tissue non-specific isoenzyme of alkaline phosphatase (TNSALP), resulting in the extracellular accumulation of its substrates - inorganic pyrophosphate (PPi), pyridoxal-5-phosphate (PLP) and phosphoethanolamine (PEA) [1]. Perinatal HPP, which manifests *in utero *and is apparent at birth, is the most severe form of HPP, with severe skeletal hypomineralization characterized by respiratory failure due to gracile, thin, short ribs, small chest, hypoplastic lungs and vitamin B6-dependent seizures [2]. Infantile HPP, which is identified in patients before 6 months of age, is characterized by failure to thrive, hypercalcemia, short limbs, limb deformities with abnormal metaphyses, small thoraces with gracile, thin ribs, respiratory complications and vitamin B6-dependent seizures [2]. Patients with perinatal or infantile HPP and vitamin B6-dependent seizures have high morbidity and mortality in the first 5 years of life [3]; prior to the availability of asfotase alfa (Strensiq®), the only enzyme replacement therapy approved for the treatment of patients with perinatal/infantile- and juvenile-onset HPP, only 42% of infants with HPP survived their first year and only 27% survived to the age of 5 years, with death typically resulting from respiratory complications [2, 4–6]. While asfotase alfa has been shown to improve survival in patients with perinatal and infantile HPP substantially [4], the characterization and management of respiratory complications in these patients remains key to their survival and to optimizing their treatment and clinical outcomes.

Multiple factors contribute to respiratory difficulties in infants with HPP. These include abnormal chest compliance arising from rachitic chest wall deformities and fractures, hypoplastic lungs, muscle weakness, episodic seizures, increased predisposition to infections secondary to TNSALP deficiency in leukocytes and, in some cases, severe tracheobronchomalacia (TBM) [4, 7–9]. Severe TBM is associated with an imminent risk of death from respiratory failure, complicated pulmonary infections and life-threatening cardiopulmonary arrests; infants with TBM therefore require intensive respiratory support from a broad group of specialists [10].

Even though TBM has been reported in patients with HPP [7–9], the clinical features, investigations and management in HPP have previously not been characterized. Here, we present a case series of five infants with perinatal HPP who had TBM contributing to severe respiratory compromise. To explore how common TBM may be amongst patients with HPP, we also reviewed respiratory function data (e.g. requirements for high positive end-expiratory pressure [PEEP] and/or peak inspiratory pressure [PIP]) in patients with perinatal and infantile HPP enrolled in the clinical trial programs for asfotase alfa.

## Patients and methods

### Case series

**Fig. 1:**
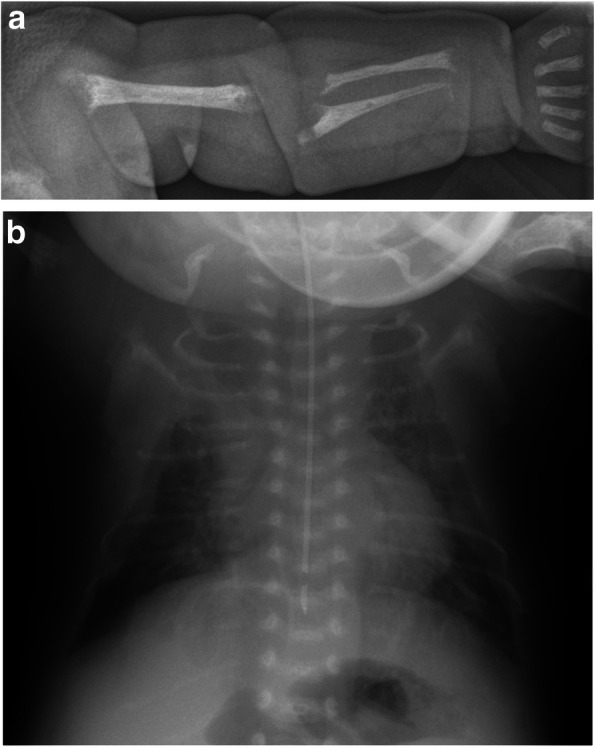
Representative radiographs (Patient 1 at age 4 weeks). **a** Radiograph of the left upper limb showing severe rickets (bold arrows) and tongue-like metaphyseal lucencies (dotted arrows). **b** Radiograph of the chest showing gracile undermineralized ribs, a narrow thoracic cavity and small lung volumes

HPP diagnosis was confirmed in five infants by physical examination, skeletal survey (Fig. 1), serum biochemistry analysis (i.e. levels of the TNSALP substrates PPi, PLP and PEA) and genetic testing. Three patients were treated at the Royal Manchester Children’s Hospital, UK, and two patients were treated at the Cincinnati Children’s Hospital Medical Center, USA. Clinical suspicions of TBM in these five patients were raised following episodes of profound desaturations and bradycardia (especially on handling) and subsequent transient/episodic increases in ventilator requirements. Diagnosis of TBM was confirmed by direct laryngotracheobronchoscopy (DLTB) or flexible bronchoscopy. In this case series, respiratory support requirements were documented and defined as invasive ventilation (by continuous positive airway pressure [CPAP] or bilevel positive airway pressure support) via endotracheal tube or tracheostomy. Patients’ demographic, genetic and clinical characteristics were summarized descriptively, as were their treatment and outcomes.

### PEEP data from clinical trials

PEEP values from two open-label, multicenter, phase 2, interventional trials of asfotase alfa in infants and young children with HPP (ENB-002-08 [NCT00744042]/ENB-003-08 [NCT01205152] and ENB-010-10 [NCT01176266]) were analyzed (excluding those patients who were included in the case series described above). These studies and the results for their pre-specified efficacy and safety outcome measures are described elsewhere [4, 11]. Briefly, 11 participants who were ≤36 months old at initial enrollment and who had a documented diagnosis of severe HPP were recruited for ENB-002-08/ENB-003-08; for ENB-010-10, 69 participants aged ≤5 years old, who had a documented diagnosis of HPP and onset of symptoms prior to 6 months of age, were recruited.

A subset of patients were identified who required any respiratory support, with PEEP ≥6 cm H_2_O and/or PIP ≥18 cm H_2_O (in line with the PEEP and PIP values measured in the patients with confirmed TBM included in the case series) at any time during the study, but who did not have a diagnosis of TBM. PEEP values from this subset of patients were examined across the course of the studies to evaluate how frequently high ventilator settings were required as well as to look at variations in the evolution of respiratory function over time, to gain insights on which infants with HPP with respiratory requirements should be screened for possible TBM. The Kendall rank correlation coefficient was used to measure ordinal associations between PEEP requirements (median and maximum) and the percentage of the duration of respiratory support for which PEEP ≥6 cm H_2_O was required.

## Results

### Case series

Five infants (three females and two males) with HPP, all of whom experienced respiratory distress at birth and required respiratory support, had TBM confirmed at ≤5 months of age. Their baseline demographic and clinical characteristics at birth and results of their laboratory analyses are presented in Tables 1 and 2, respectively. *ALPL* gene mutations were found in all five patients; two infants had compound heterozygous gene mutations and the other three had homozygous mutations. All mutations except the c.876_872delAGGGGACinsT mutation identified in Patient 3 have been reported previously [12, 13].

**Table 1:** Baseline demographic and clinical characteristics at birth in infants included in the case series

Characteristic	Patients Who Received Asfotase Alfa as Part of the ENB-010-10 Study			Patient Who Received Asfotase Alfa Through Compassionate-Use Program	Patient Who Received Asfotase Alfa as Licensed Medication Post-Authorization
	Patient 1	Patient 2	Patient 3	Patient 4	Patient 5
Sex	Male	Male	Female	Female	Female
Ethnicity	White Caucasian	White Caucasian	White Caucasian	Asian-Pakistani	White Caucasian
Gestational age	Term	Term	35 weeks, 4 days	34 weeks	Term
Birth weight	2.89 kg	3.46 kg	3.06 kg	1.69 kg	3.46 kg
Post-birth respiratory support	CPAP ventilation at birth; intubation and ventilation starting at 4 weeks	Intubation and ventilation	Intubation and ventilation	Intubation and ventilation: surfactant	Intubation and ventilation at birth
Presence of skeletal manifestations of HPP	Yes	Yes	Yes	Yes	Yes
Presence of hypotonia	Yes	Yes	Yes	Yes	Yes
Presence of feeding difficulty	Yes	Yes	Yes	Yes	Yes

*CPAP* Continuous positive airway pressure, *HPP* Hypophosphatasia

**Table 2:** Results of laboratory analyses conducted for infants included in the case series

Laboratory Measure	Patients Who Received Asfotase Alfa as Part of the ENB-010-10 Study			Patient Who Received Asfotase Alfa Through Compassionate-Use Program	Patient Who Received Asfotase Alfa as Licensed Medication Post-Authorization
	Patient 1	Patient 2	Patient 3	Patient 4	Patient 5
Alkaline phosphatase at birth (normal range: 82–383)	Undetectably low	14 U/L	18 U/L	Undetectably low	<5 U/L
Calcium (normal range: 2.25–2.74)	3.0 mmol/L at birth	2.9 mmol/L at 4 weeks	1.3 mmol/L (ionized, normal range: 0.85–1.45) at 8 days	2.9 mmol/L at 4 weeks	3.0 mmol/L at birth
Phosphorus (normal range)	NA	4.3 mg/dL (2.5–4.5) at 4 weeks	6.8 mg/dL (2.5–4.5) at 8 days	2.4 mmol/L (1.8–2.3) at 4 weeks	2.3 mmol/L (1.8–2.3) at birth
PLP (B6) (normal range)	NA	>2000 ng/mL (11.8–68.3) at 1 day	NA; PEA 413 μmol/L (0–300)	4740 ng/mL (11.8–68.3) at 4 weeks	3940 nmol/L (20–140)
PPi (normal range: 1.33–5.71)	9.5 μmol/L at 4 weeks	10.4 μmol/L at 5 weeks	7.4 μmol/L at 7 weeks	9.5 μmol/L at 4 weeks	NA
*ALPL* mutation	Homozygous: c.147 G > A, (p.Gly491Arg) secondary to uniparental disomy [12]	Compound heterozygous: c.668 G > A (p.Arg223Gln) and c.1171 C > T (p.Arg391Cys)	Compound heterozygous: c.876_872delAGGGGACinsT and c.650 T > C (p.Val217Ala)	Homozygous: c.1336 G > A (p.Ala466Thr)	Homozygous: c.400_401delinsCA, (p.Thr134His)

*NA* Not available, *PEA* Phosphoethanolamine, *PLP* Pyridoxal-5-phosphate, *PPi* Inorganic pyrophosphate

### Case report findings

#### Patient 1

##### HPP medical history and management

Patient 1 presented immediately after birth with poor feeding, significant hypotonia and respiratory distress. Antenatal scans showed shortening of the long bones and a skeletal survey revealed manifestations of HPP with severe rickets, thin undermineralized ribs, a small thoracic cavity and small lung volumes (Fig. 1). The patient was included in study ENB-010-10 and treatment with asfotase alfa was commenced at 1 month of age.

##### Respiratory measures and mechanics

The patient experienced progressive worsening of respiratory function and continued to have episodes of desaturations, requiring PEEP of 8 cm H_2_O. A DLTB performed at 2 months of age revealed severe laryngotracheobronchomalacia, which required PEEP of 12 cm H_2_O to keep the airways patent, with positive pressure ventilation delivered through tracheostomy. Respiratory function subsequently improved, with reduction in PEEP to 6 cm H_2_O at 9 months of age.

##### Follow-up

**Fig. 2:**
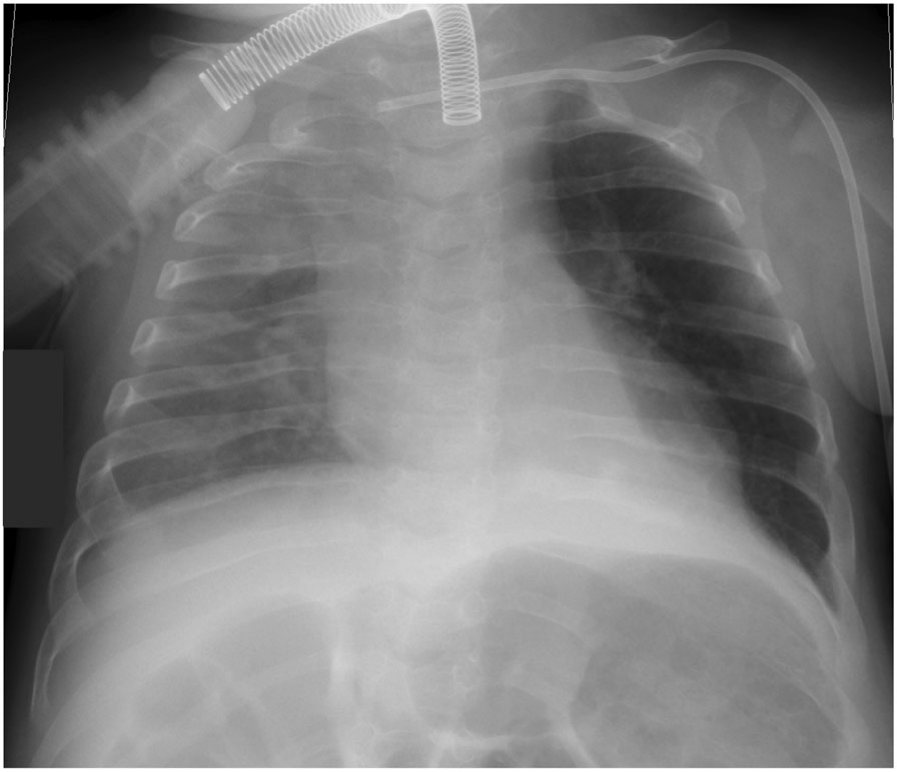
Chest radiograph of Patient 1 after 11 months of treatment with asfotase alfa. Chest radiograph of Patient 1 at 1 year of age, after 11 months of treatment with asfotase alfa. The radiograph showed an improvement in the mineralization of the ribs and an increase in the volume of the thoracic cavity compared with that observed at age 4 weeks (see Fig. 1b)

At 1 year of age, ventilation was tapered and changed to CPAP via tracheostomy; chest radiograph showed significantly improved mineralization of the ribs and increased lung volume (Fig. 2). By 14 months of age the patient was self-ventilating in room air with complete clinical resolution of laryngotracheobronchomalacia.

#### Patient 2

##### HPP medical history and management

Radiographs taken at 1 day of age revealed that Patient 2 had remarkably diminished ossification of the skull with almost no cranial calcification. He also had diminished ossification and height of vertebral bodies, and absent ossification of the humeral, radial and ulnar metaphyses with marked metaphyseal irregularity, fragmentation and fraying. His chest was small and the bones abnormal, with absent ossification of medial ribs and gracile appearance of the ribs. The patient was included in study ENB-010-10, and treatment with asfotase alfa was initiated at 5 weeks of age.

##### Respiratory measures and mechanics

**Fig. 3:**
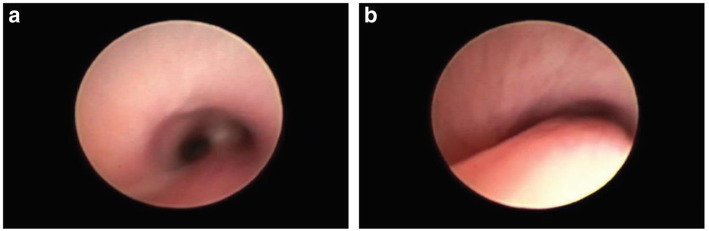
Representative tracheobronchoscopy (Patient 2 at age 5 months). Representative images of the right mainstem bronchus during inspiration (**a**) and expiration (**b**) from Patient 2

At 1 month of age, the patient required PEEP of 8 cm H_2_O. From 6 weeks of age, continuous ventilator support was required with PEEP of up to 12 cm H_2_O. Diagnosis of TBM was confirmed by tracheobronchoscopy at 5 months of age (Fig. 3).

##### Follow-up

There was significant improvement at 12 months of age, with mild TBM and episodes of respiratory arrest resolved; PEEP of 10 cm H_2_O was required. At 17 months of age, the lower airways appeared normal, with no appreciable dynamic collapse during breathing at rest; however, there was profound TBM when the patient was coughing or during forced exhalation, and PEEP of 10 cm H_2_O was still required. At 5 years and 9 months of age, the patient was off the ventilator for 4 h three times per day, and discontinuation of daytime ventilation was planned for the following months. There were no serious setbacks, and, despite global developmental delays, language was improving and the patient was able to recognize most objects and animals.

#### Patient 3

##### HPP medical history and management

Initial examination of Patient 3 found a very large anterior fontanelle, cranial molding, small chest with subcostal retractions and short, deformed extremities with bowing (equinovarus). Her feet were angulated and clubbed, with dimpling below the knees. X-rays showed severely decreased mineralization, diffuse osteopenia, poorly ossified ribs and irregularity of the right proximal humerus consistent with fracture. Additional radiographs documented bilateral humeral fractures. The patient was included in study ENB-010-10, and treatment with asfotase alfa was initiated at 1 month and 23 days of age.

##### Respiratory measures and mechanics

At 1 month and 19 days of age, there was profound dynamic collapse of the trachea and bronchi during coughing or heavy breathing, even while intubated and receiving positive pressure. Despite increasing PEEP to 10 cm H_2_O, respiratory arrests with bradycardia requiring major intervention (100% fraction of inspired O_2_ delivered by positive pressure, and chest compressions for bradycardia) occurred on an almost weekly basis. At 5 months of age, there was profound bronchomalacia with complete collapse of the airway lumen on coughing or heavy breathing.

##### Follow-up

The severe respiratory episodes decreased in frequency and at 12 months of age flexible bronchoscopy revealed moderate TBM, which, though considered significant, appeared to be improved from the previous evaluation at 6 months of age, with the PEEP requirement decreased to 9 cm H_2_O from 12 cm H_2_O. At 18 months of age, there remained significant TBM, and PEEP of 12 cm H_2_O was required. At 5 years and 5 months of age, the patient was off the ventilator during the daytime, with rapid and complete discontinuation of ventilation and microlaryngobronchoscopy planned. In addition, cognitive development was progressing and the patient was able to recognize all letters, numbers, animals and body parts. The patient was also able to stand unassisted for short periods.

#### Patient 4

##### HPP medical history and management

Patient 4 was found to have dysmorphic features with short limbs, craniotabes and significant hypotonia, and her skeletal survey revealed characteristic features of HPP. Treatment with asfotase alfa was commenced at 1 month of age through a compassionate-use program.

##### Respiratory measures and mechanics

Despite improvements in biochemistry results within 7 days of starting treatment with asfotase alfa, Patient 4 continued to have increased ventilator requirements (including PEEP of up to 6 cm H_2_O) with episodes of significant bradycardia and desaturations. At 3 months and 9 days of age, these culminated in an acute deterioration requiring chest compression and inotropes for cardiac arrest. The dose of asfotase alfa was then increased and within 2 weeks she showed improvement. A DLTB performed at 5 months of age demonstrated significant laryngotracheobronchomalacia, with an opening pressure (PEEP) of 12 cm H_2_O.

##### Follow-up

Respiratory function significantly improved with the increase in PEEP, and by 8 months of age PEEP was reduced to 9 cm H_2_O. At 11 months of age, a repeat DLTB showed complete resolution of laryngotracheobronchomalacia, and by 2 years of age the patient had been weaned off ventilation completely.

### Patient 5

#### HPP medical history and management

Patient 5 was diagnosed with HPP antenatally at 32 weeks of gestational age when a fetal ultrasound scan showed skeletal features suggestive of HPP. Her mother previously underwent a termination of a fetus that had HPP confirmed following genetic testing, and both parents were known to be heterozygous carriers for *ALPL* gene mutation. Treatment with asfotase alfa was initiated immediately after birth.

#### Respiratory measures and mechanics

Patient 5 was intubated and ventilated at birth. Her ventilatory requirements increased at 3 months of age, with progressive increase in fraction of inspired oxygen from 21% to 50–60% and of PIP from 16 to 22 cm H_2_O with episodic worsening during routine care and handling. A DLTB at 3 months showed TBM, which required PEEP of 12 cm H_2_O to keep the airway patent.

#### Follow-up

High PEEP (up to 12 cm H_2_O) was required by Patient 5 until 6 months of age, when it was reduced to 6 cm H_2_O. Weaning from ventilation was then initiated and complete by 9 months of age. At 13 months of age, a repeat DLTB showed residual TBM, but this was not clinically significant as the patient no longer required ventilatory support.

In summary, all patients required tracheostomy for long-term ventilation with PEEP of up to 12 cm H_2_O following commencement of respiratory support at birth. Images from one patient at age 5 months show the right mainstem bronchus during inspiration (Fig. 3a) and expiration (Fig. 3b) (the source video file is included in the supplemental materials). Normal anatomy and airway patency are evident during inspiration; on expiration, however, near-complete collapse of the right mainstem bronchus is seen.

**Additional file 1.**

All patients received ongoing treatment with asfotase alfa 6–15 mg/kg/week, starting at 0–7 weeks of age. TBM was confirmed within 6 months of birth for all five patients and was suspected in one patient as early as 8 weeks after birth. All patients had frequent episodes of profound desaturations and bradycardia, and three experienced cardiorespiratory arrests. Treatment and outcomes are summarized for each patient in Table 3.

**Table 3:** Overview of treatment and patient outcomes in infants included in the case series

Treatment	Patients Who Received Asfotase Alfa as Part of the ENB-010-10 Study			Patient Who Received Asfotase Alfa Through Compassionate-Use Program	Patient Who Received Asfotase Alfa as Licensed Medication Post-Authorization
	Patient 1	Patient 2	Patient 3	Patient 4	Patient 5
Asfotase alfa dosage, mg/kg/week	1 month: 6 3 months: 9 Current: 7.5	5 weeks: 6 6 months: 7.8 9 months: 7.5 Current: 9	7.5 weeks: 6 Current: 12	1 month: 6 3.5 months (post-cardiac arrest): 15 Current: 7.5	Birth: 6 2 months: 9 3 months: 15 Current: 7.5
Surgical treatments	5 weeks: tracheostomy	6 weeks: tracheostomy 4 months: gastrostomy	7 weeks: tracheostomy 3 months: gastrostomy	6 weeks: tracheostomy 1 year: gastrostomy	3 days: tracheostomy and central venous access 16 months: left ureteroscopy with laser lithotripsy for renal stone obstructing left ureter causing left hydronephrosis 19 months: closure of tracheostomy 2 years: surgical correction of craniosynostosis
Age at TBM diagnosis	2 months	5 months	5 months (suspected at 8 weeks)	5 months	3 months
Current age	5 years, 8 months	5 years, 9 months	5 years, 5 months	7 years, 2 months	3 years, 4 months
Long-term follow-up and current status	15 months: complete clinical resolution and self-ventilating in room air	17 months: normal-appearing lower airways, but profound TBM when coughing or bearing down; BiPAP: PIP 24 cm H_2_O, PEEP 10 cm H_2_O 27 months: remains on ventilator; respiratory issues (viral infections) requiring hospital readmission; BiPAP: PIP 22 cm H_2_O, PEEP 8 cm H_2_O 5 years, 9 months: off ventilator for 4 h three times daily; plan to completely wean from daytime ventilator by 6 years of age	18 months: significant TBM identified (severity difficult to assess owing to well-positioned custom tracheostomy tube in distal trachea); BiPAP: PIP 26 cm H_2_O, PEEP 12 cm H_2_O 23 months: tracheostomy in situ with ventilator support; BiPAP: PIP 25 cm H_2_O, PEEP 12 cm H_2_O 5 years, 5 months: off ventilator for 16–18 h daily; plan to rapidly completely discontinue ventilator support in the coming months	2 years: all ventilator support removed	9 months: all ventilator support removed

*BiPAP* Bilevel positive airway pressure, *PEEP* Positive end-expiratory pressure, *PIP* Positive inspiratory pressure, *TBM* Tracheobronchomalacia

### PEEP data from clinical trials

Among all infants who need ventilatory support, PEEP requirements and ventilation strategies are heavily influenced by the underlying cause of chronic respiratory failure. However, in our experience, infants without significant lung disease or TBM may typically require PEEP of 4–8 cm H_2_O. Of the 80 patients included in the asfotase alfa clinical trials, 39 required respiratory support at any time; 23 of these 39 (59%) patients required high-pressure respiratory support (PEEP ≥6 cm H_2_O and/or PIP ≥18 cm H_2_O) via any route, at any time (22 of these patients required respiratory support at baseline, and one patient did not have baseline respiratory support information). Three of these patients are included in the case series described above, and therefore not included in these additional analyses. Thus, results of an analysis of PEEP measurements (in line with the requirements of the case series patients) for 20 patients are presented here and in Supplemental Table 1. The median PEEP at any time was 8 cm H_2_O, with a highest PEEP of 20 cm H_2_O. The median number of days of high-pressure respiratory support for these patients during the studies was 176 (1–1065; the median number of days on study for these patients was 912.5 [2–2724]), or 85.2% (9.3–100.0%) of the duration of respiratory support. Most of these 20 patients required high-pressure respiratory support across the entire period of their respiratory support. However, there was no correlation between the duration that those patients received ventilatory support and a PEEP requirement of ≥6 cm H_2_O (median PEEP τ = .15, *P* = .44; maximum PEEP τ = .09, *P* = .65). In this cohort, there were seven patients with even higher respiratory requirements for PEEP (≥8 cm H_2_O) and four patients with both PEEP ≥8 cm H_2_O and PIP ≥20 cm H_2_O at any time. There was a high degree of heterogeneity in PEEP requirements across the cohort of included patients; however, six patients presented with consistently high PEEP values (≥8 cm H_2_O) over periods of more than 6 months.

## Discussion

We have detailed the clinical course of TBM in five patients with perinatal-onset HPP; in our cohort, TBM contributed significantly to respiratory morbidity. More specifically, these patients required prolonged respiratory support with high PEEP in an intensive care unit setting and ongoing positive pressure to prevent collapse of airways. We typically increase PEEP in response to patient behaviors that cause us to suspect TBM. However, in many cases of severe TBM, the patient can overcome the distending pressure of even very high PEEP through coughing or the Valsalva maneuver. Heavy breathing may occur if a child is upset or crying. Their peak inspiratory flow rate rises, causing airflow to become more turbulent. It is the loss of laminar airflow that contributes to the softened airway collapsing. Other patients with severe TBM may have increased work of breathing at baseline during tidal breathing and will have improved work of breathing as PEEP is increased. Patients presenting with severe oxygen desaturation and decreased heart rate often respond to positive pressure bagging with 100% oxygen, with chest compressions also required on some occasions. Based on our findings, infants with perinatal HPP who have episodes of oxygen desaturation and/or changes in ventilator settings, especially increased PEEP, should be suspected of having TBM and investigated and managed accordingly.

Data from the clinical trial program for asfotase alfa provided the opportunity to evaluate a larger population of patients with HPP who required high-pressure respiratory support. Of the 39 infants with HPP who required respiratory support, 51% (20/39) who did not have TBM as a reported adverse event required high-pressure respiratory support (PEEP ≥6 cm H_2_O and/or PIP ≥18 cm H_2_O) during the studies. PEEP requirements were highly variable across the population, ranging up to 20 cm H_2_O, with 18% of patients (7/39) requiring PEEP of ≥8 cm H_2_O. It is possible that TBM contributed to the respiratory complications in some of these patients. It is important to note that factors other than TBM, such as chest deformity, restricted respiratory function, muscle weakness or sepsis, may lead to high PEEP requirements in infants with HPP. Furthermore, it is possible that infants with HPP requiring PEEP of <6 cm H_2_O may have TBM, although it is not likely to be clinically significant. Therefore, PEEP measurements cannot be used retrospectively to infer TBM or other specific respiratory symptoms or complications of HPP. However, given the frequency of high PEEP measures in the patients included in the case series, who had severe HPP and TBM, physicians should be aware that this may reflect respiratory complications including, although not limited to, TBM.

There is considerable variation in PEEP strategies employed in different pediatric intensive care units. Although bronchoscopy may inform decisions about initial respiratory requirements during quiet spontaneous respiration, these may be insufficient when a child is crying or coughing or has an intercurrent lower respiratory tract infection. Furthermore, the weaning of PEEP as symptoms improve is often empirical. Consequently, it is difficult to draw firm conclusions about either the severity of TBM or the duration of treatment solely from PEEP data. However, these data do alert us to identifying children who appear to be dependent on externally applied pressure.

Diagnosis of TBM is multifaceted [14] but should be confirmed with DLTB or flexible bronchoscopy, although bronchoscopic diagnosis of TBM is highly dependent on the conditions under which the procedure is performed. For example, if a patient is heavily sedated or paralyzed, sufficient airway collapse may not be noted if the patient does not cough or perform a Valsalva maneuver. Very severe TBM may be seen with tidal breathing, but the vigor of respiratory effort can still impact diagnosis. Earlier diagnosis and multidisciplinary approaches to care are likely to lead to improved outcomes for patients with HPP and associated TBM; consultation with a pediatric pulmonologist and a pediatric otolaryngologist is recommended for the management of such cases.

The effectiveness of asfotase alfa treatment in patients with HPP and TBM was not evaluated in the case series or the analysis of clinical trial data reported here. Decreased ventilator requirements following asfotase alfa treatment are in line with previously reported results in a case study of respiratory mechanics in an infant with perinatal HPP [9]. However, it would be difficult to determine whether any improvements in TBM were directly attributable to the promotion of skeletal mineralization by asfotase alfa or to gradual improvements in airway lumen and cartilage rigidity that may have occurred naturally with age [15]. In the latter case, the increased survival associated with asfotase alfa likely provided benefit in allowing time for the airways to mature.

This report does not evaluate adjustments in asfotase alfa dosing required to manage TBM and high respiratory support requirements in patients with perinatal HPP. All five infants included in the case series were, at times, treated with doses higher than the standard asfotase alfa dose of 6 mg/kg/week (range: 9–15 mg/kg/week, on the basis of skeletal mineralization and growth), and the dosage was escalated in response to high ventilatory requirements. It is not possible to determine whether dose escalation contributed to improvements in TBM. Therefore, all patients with similar clinical characteristics should be managed on the standard dose of asfotase alfa, and any plan to increase the dose should be discussed with medical experts worldwide who have experience managing perinatal HPP, especially in the face of respiratory failure.

## Conclusion

The findings of the current study show that TBM may be a more common comorbidity of HPP than previously thought and is probably an under-recognized contributing factor in respiratory failure in infants with HPP. It is important to screen for potential TBM in infants with HPP presenting with persistent respiratory complications (including increased PEEP requirements, oxygen desaturation events or repeated failed extubations), by using DLTB or flexible bronchoscopy at an early stage, and to initiate appropriate management strategies and treatment, as assessed by a specialist clinical team, to ensure the best prognosis.

## Supplementary information

**Additional file 2: Supplemental table 1.** Infants included in the clinical trials for asfotase alfa who required high-pressure respiratory support.

## Data Availability

Qualified academic investigators may request participant-level, de-identified clinical data and supporting documents (statistical analysis plan and protocol) pertaining to this study. Further details regarding data availability, instructions for requesting information, and the data disclosure policy will be available on the Alexion.com website (http://alexion.com/research-development).

## Abbreviations

CPAP: Continuous positive airway pressure
DLTB: Direct laryngotracheobronchoscopy
HPP: Hypophosphatasia
PEA: Phosphoethanolamine
PEEP: Positive end-expiratory pressure
PIP: Peak inspiratory pressure
PLP: Pyridoxal-5-phosphate
PPi: Inorganic pyrophosphate
TBM: Tracheobronchomalacia
TNSALP: Tissue non-specific isoenzyme of alkaline phosphatase
