# Supplementary material for: Characterization of tracheobronchomalacia in infants with hypophosphatasia
Source: Orphanet J Rare Dis. 2020 Aug 6;15:204. doi: 10.1186/s13023-020-01483-9 (PMC7407429; doi:10.1186/s13023-020-01483-9)
Supplement: Supplementary file 2 — Additional file 2: Supplemental table 1. Infants included in the clinical trials for asfotase alfa who required high-pressure respiratory support. [file 13023_2020_1483_MOESM2_ESM.docx]

**SUPPLEMENTAL TABLE 1** **INFANTS INCLUDED IN THE CLINICAL TRIALS FOR ASFOTASE ALFA WHO REQUIRED HIGH-PRESSURE RESPIRATORY SUPPORT**

| Parameter | Number of Patients | Mean | SD | Median | Minimum | Maximum |
| --- | --- | --- | --- | --- | --- | --- |
| Duration of study, days | 20 | 1040.7 | 951.0 | 912.5 | 2.0 | 2724.0 |
| Number of days of respiratory support during study, per patient | 20 | 374.0 | 428.1 | 254.1 | 1.0 | 1604.0 |
| Percent of time on respiratory support during study, per patient | 20 | 54.4 | 36.6 | 46.4 | 3.5 | 99.9 |
| Number of days on which PEEP requirement was ≥6 cm H_2_O during study, per patient | 18 | 247.0 | 259.2 | 176.0 | 1.0 | 1065.5 |
| Percent of time receiving respiratory support during which PEEP requirement was ≥6 cm H_2_O, per patient | 18 | 73.6 | 30.9 | 85.2 | 9.3 | 100.0 |

Summary of respiratory support provided to infants with perinatal HPP included in the clinical trial program for asfotase alfa who required respiratory support and PEEP ≥6 cm H_2_O and/or PIP ≥18 cm H_2_O at any time on study

HPP, hypophosphatasia; PEEP, positive end-expiratory pressure; PIP, positive inspiratory pressure; SD, standard deviation.
